# Inherited epidermolysis bullosa and squamous cell carcinoma: a systematic review of 117 cases

**DOI:** 10.1186/s13023-016-0489-9

**Published:** 2016-08-20

**Authors:** H. Montaudié, C. Chiaverini, E. Sbidian, A. Charlesworth, J-P. Lacour

**Affiliations:** 1 Department of Dermatology, University Hospital of Nice, 151 route de Saint Antoine de Ginestière, Hôpital Archet 2, 06200 Nice, France; 2 Reference Centre for Hereditary Epidermolysis Bullosa, University Hospital of Nice, Nice, France; 3 Department of Dermatology, Henri Mondor Hospital, INSERM, Centre d’Investigation Clinique, Créteil, France

**Keywords:** Cutaneous squamous cell carcinoma, Inherited epidermolysis bullosa, Systematic review, Chemotherapy, Target therapy, Radiotherapy

## Abstract

**Background:**

Inherited epidermolysis bullosa (EB) comprises a highly heterogeneous group of rare diseases characterized by exacerbated skin and/or mucosal fragility and blister formation after minor mechanical trauma. Level of cleavage in the skin, clinical features with immunofluorescence antigen mapping and/or electron microscopy examination of a skin biopsy and/or gene involved, type(s) of mutation present and sometimes specific mutation(s), allow to define the EB type and subtype. This family of genodermatoses exposes patients to several complications, cutaneous squamous cell carcinoma (cSCC) being the most severe of them.

**Objective:**

The aim of this systematic review was to document patients with EB who developed cSCC.

**Methods:**

A systematic literature search was performed, from inception to March 2014, using Medline, Embase, Cochrane and ClinicalTrials.gov databases. Only articles published in English and French were selected. The diagnosis of EB had to be confirmed by EM and/or IFM and/or mutation analysis, while cSCC had to be confirmed by histological analysis.

**Results:**

Of 167 references in the original search, 69 relevant articles were identified, representing 117 cases. cSCCs were identified in all types of EB, though predominantly in recessive dystrophic EB (RDEB) forms (81 cases (69.2 %)). The median age at diagnosis was 36 years old (interquartile range (IQR), 27-48 years and range, 6-71 years) for all forms. Of those with measurements in the literature (88 cases (75.2 %)), tumor size was greater than 2 centimeters in 52 cases (59.1 %). The histopathological characteristics were specified in 88 cases (75.2 %) and well-differentiated forms predominated (73.9 %). No conclusion could be drawn on the choice of surgical treatment or the management in advanced forms.

**Limitations:**

This study was retrospective and statistical analysis was not included due to various biases. This study design did not allow to infer prevalence, nor EB subtype risk for cSCC occurrence.

**Conclusions:**

Our study correlated with historical data shows that most of the cSCCs occurred in subjects with the RDEB subtype, however reports also show that cSCCs can present in any patients with EB. The first signs of cSCC developed at a younger age in EB patients than in non-EB patients. Interestingly, the cSCC duration, before its diagnosis, was shorter in individuals with RDEB than with junctional EB (JEB) and dominant dystrophic EB (DDEB).

This study further emphasizes the importance of regular monitoring of EB patients, particularly with the RDEB subtype as they developed cSCC at a younger age.

## Background

Epidermolysis bullosa (EB) is an inherited skin disorder characterized by exacerbated skin and/or mucosal fragility and blister formation after minor mechanical trauma.

Depending on the level of cleavage in the skin, four major types of EB can be distinguished by immunofluorescence antigen mapping (IFM) and/or transmission electron microscopy (EM): EB simplex (EBS), junctional EB (JEB), dystrophic EB (DEB), and Kindler syndrome (KS). There is extensive phenotypic variability leading to more than 30 phenotypic subtypes. A new classification has recently been proposed by *Fine* et al. for EB patients [1]. These patients are exposed to many complications, including nutritional and infectious problems [2]. The most common evolution and cause of death in these patients is cutaneous squamous cell carcinoma (cSCC), especially for patients with recessive dystrophic EB (RDEB), where cSCC generally becomes very aggressive and thus yields a poor prognosis [3–5]. Although the occurrence of cSCC in EB is well known, the risk depending on the type of EB, the prognosis, the clinical and histological features as well as the methods of its management are poorly documented. A review was published in 2002 [4], but methodology was not described and new cases and technologies have appeared since then. Very recently, clinical practice guidelines for the management of cSCC in patients with EB have been published [6]. These recommendations have been drawn up from a systematic review of the available literature which has not been reported in detail.

The objective of our study was to review all cases of EB published in the literature to better characterize cSCC associated with EB.

## Material and methods

We performed a systematic review of all studies reporting or investigating the association of EB with cSCC.

Medline, Embase, Cochrane Central Register and ClinicalTrials.gov databases were systematically searched. Data from registries were not collected. An expert in the field, and member of the French group “Association Recommandations en Dermatologie (aRED)”, oversaw our activities to ensure that no relevant studies were missed. We used a combination of Medical Subject Headings (MeSH) for our search. The search terms, listed in Table 1, were defined with a librarian member of the aRED group. We limited the literature search to articles in English or French. No restrictions concerning the age or sex of the patients or date of publication were imposed. Full copies of the relevant papers were obtained. The diagnosis of EB had to be confirmed by EM and/or IFM and/or mutation analysis. Because most cases were reported before the updated classification [1] we did not use the latest recommended terminology. The diagnosis of cSCC had to be confirmed by histological analysis.

**Table 1:** Search strategy used for Medline/Embase/Cochrane Library/ClinicalTrials.gov in our systematic review

“Carcinoma, Squamous Cell”[Mesh]	
OR	
“Bowen’s Disease”[Mesh]	
OR	
“Neoplasms, Squamous Cell”[Mesh:noexp]	
OR	
“Acanthoma”[Mesh]	
OR	
“Carcinoma, Papillary”[Mesh]	
OR	
“Carcinoma, Verrucous”[Mesh:noexp]	
OR	
“Carcinoma, Adenosquamous”[Mesh]	
OR	
“Carcinoma in Situ”[Mesh]	
OR	
“Cervical Intraepithelial Neoplasia”[Mesh]	
OR	
“Prostatic Intraepithelial Neoplasia”[Mesh]	
OR	
“Squamous Cell Carcinoma”[title/abstract]	
AND	
(“Epidermolysis Bullosa”[Mesh:noexp]	
OR	
“Epidermolysis Bullosa Dystrophica”[Mesh]	
OR	
“Epidermolysis Bullosa, Junctional”[Mesh]	
OR	
“Epidermolysis Bullosa Simplex”[Mesh]	
OR	
“Poikiloderma of Kindler”[Supplementary Concept]	
OR	
“Epidermolysis Bullosa”[title/abstract]	
OR	
“Kindler syndrome”[title/abstract])	
AND (English[lang]	
OR	
French[lang])	

The following data were collected from the articles: 1. sex; 2. race/ethnicity categorized into North African, Caucasian, Asian and Hispanic; 3. Fitzpatrick skin type; 4. inbreeding; 5. family history of cSCC; 6. age at diagnosis of cSCC; 7. type and subtype of EB based upon IF, EM and genetic sequencing data; 8. clinical features of cSCC: localization (upper limb, lower limb, trunk, back, buttocks, and mucosa); clinical presentation of the tumor(s) (erosive, ulcerated, crusted, exophyting, hyperkeratotic); size of tumor(s) (≤2 cm, > 2 cm, > 5 cm); type of EB lesion on which the cSCC occurred (chronic blistering, atrophic scarring or healthy skin); 9. histological features: degree of differentiation (well differentiated, moderately differentiated, poorly differentiated, not specified); depth (dermis, subcutaneous fat, fascia, muscle, cartilage, bone, other, unknown); perineural invasion, lymphatic vessel invasion; 10. stage and evolution of disease (localized, regional or metastatic disease; alive or demise due to cSCC); 11. use of SLNB; use of 18 Fluorodeoxyglucose Positron Emission Tomography/computed tomography (18-FDG PET/CT) and 12 treatments (surgery, radiotherapy, systemic therapy).

### Statistical analysis

No statistical analysis, or meta-analysis, could be done due to the heterogeneity of the studies. There were many missing data for each variable and the retrospective design implicated too many biases, particularly publication and reporting biases. For these reasons, we did not incorporate statistical analysis in the study.

## Results

**Fig. 1:**
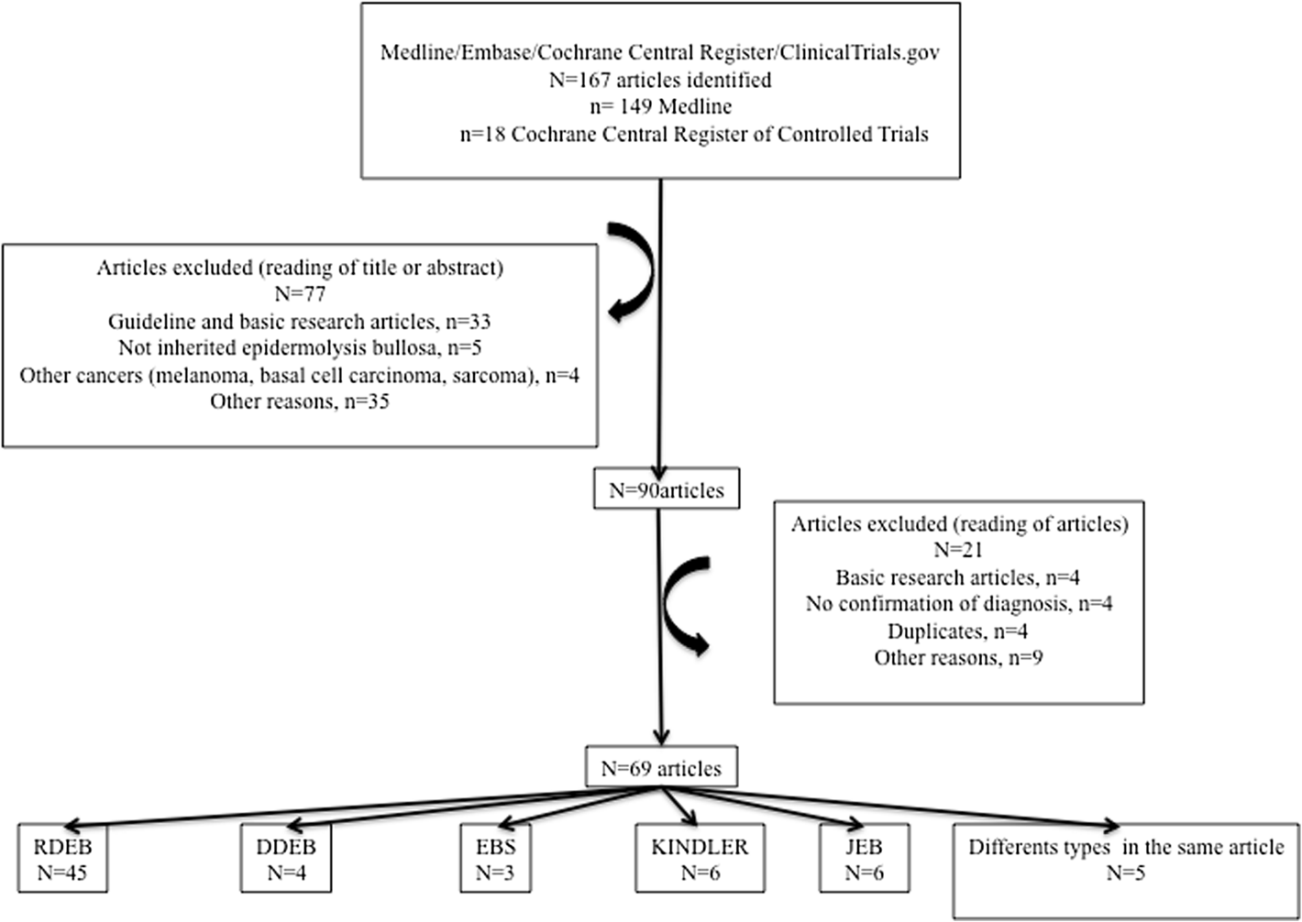
Study design flowchart. DDEB, dominant dystrophic epidermolysis bullosa; EBS, EB simplex; JEB, junctional epidermolysis bullosa; RDEB, recessive dystrophic epidermolysis bullosa

The results of the search strategy are shown in Fig. 1. Sixty-nine articles were finally selected, representing 117 cases of EB affected by at least one cSCC. Forty-five patients (38.5 %) had at least 2 cSCC; among them 36 (80.0 %) were DEB patients, 7 (15.6 %) were JEB patients, 2 (4.4 %) were KS patients, and there were no EBS patients. All forms of EB were represented; the articles concerned 45 RDEB, 4 DDEB (dominant dystrophic EB), 6 JEB, 3 EBS and 6 KS. For 5 articles, several forms of EB were included in the same article. The repartition of reported cases among different types and subtypes of EB is presented in Table 2.

**Table 2:** Repartition of reported cases among different types and subtypes of EB

Number of cases	Per type	EBS 3			JEB 19			DEB 88					KS 7
	Per subtype	EBS-DM	EBS-AR	NS	H-JEB	nH-JEB	NS	DDEB	RDEB				7
		1	1	1	0	18	1	7	81				
									RDEB- HS 20	RDEB- nHS 6	RDEB-I 2	NS 53	

*AR* autosomal recessive, *DDEB* dominant dystrophic epidermolysis bullosa, *DEB* dystrophic epidermolysis bullosa, *DM* Dowling-Meara, *EBS* EB simplex, *JEB* junctional epidermolysis bullosa, *KS* Kindler syndrome, *H* Herlitz, *HS* Hallopeau-Siemens, *nH* non-Herlitz, *nHS* non Hallopeau-Siemens, *RDEB* recessive dystrophic epidermolysis bullosa, *RDEB-I* recessive dystrophic epidermolysis bullosa-Inversa, *NS* not specified

Considering all forms of EB: 1. No sex difference was observed except for JEB with a sex ratio of 3.75:1 for men: women; 2. a higher incidence of cSCCs in Caucasians (50.4 %) was observed compared to that observed in Asians, Northern Africans and Hispanics; 3. the median age at diagnosis was 36 years old (interquartile range (IQR), 27-48 years and range, 6-71 years); 4. only 16.2 % of EB cases were confirmed by molecular analysis; 5. Fitzpatrick skin type and sun exposure were never specified; 6. Family history was specified in only 38 cases (32.5 %) and was present in 13 cases (34.2 %); 7. the majority of cSCCs occurred on lower (54.7 %) and upper extremities (30.8 %) and mucosal SCCs were described in ten cases (8.6 %); 8. clinical features were specified in 98 cases (83.8 %), the most frequent being ulcerations, in 44 cases (44.9); 9. of those reported in the literature with measurements (88 cases (75.2 %)), tumor size was greater than 2 centimeters in 52 cases (59.1 %); 10. the mean time between the occurrence of cSCC and the confirmed diagnosis from a biopsy was specified in 42 cases (35.9 %) and was 9.7 months; 11. the degree of differentiation was specified in 88 cases (75.2 %) and well-differentiated forms predominated (73.9 %); 12. metastases were specified in 88 cases (75.2 %), with 14 cases (15.9 %) and 20 cases (22.7 %) of loco-regional and visceral metastases, respectively; 13. death related to cSCC was specified in 78 cases (66.7 %) and was related to cSCC in 32 cases (41.0 %). Four patients were lost to follow-up; 14. a relapse of cSCC, was specified in 72 cases (61.5 %) and occurred in 26 cases (36.1 %); 15. time to recurrence was specified in 26 cases (22.2 %) and the mean time was 14.9 months. The detailed demographic, clinical and histopathological features of the patients are presented in Table 3.

**Table 3:** Detailed demographic, clinical and histopathological features of the patients

		All n (%)	EBS	JEB	DEB DDEB RDEB		KS
Sex	M n (%)	63 (53.8)	2	15	5	37	4
	F n (%)	54 (46.2)	1	4	2	44	3
	SR^j^ (M/F)	1.17	2	3.75	2.5	0.84	1.33
Ethnicity	Caucasian	59 (50.4)	1	16	3	36	3
	Asian	10 (8.5)	1	0	0	8	1
	North African	5 (4.3)	0	0	0	5	0
	Hispanic	1 (0.9)	0	0	0	0	1
	NS	42 (35.9)	1	3	4	32	2
Median age at diagnosis years (range), specified in 117 cases (100 %) *n* = number of patient		36 (6-71) 117	41 (39-41) 3	49 (28-71) 19	45 (34-69) 7	32.5 (6-67)^a^ 81	38.5 (16-65) 7
Genetic mutations confirmed diagnosis^b^		19 (16.2)	2	7	1	6	3
Fitzpatrick skin type and sun exposure		NS	NS	NS	NS	NS	NS
Family history of cSCC^c^	Present	13	1	4	3	4	1
	Absent	25	2	1	2	19	1
	NS	79	0	14	2	58	5
Location^d^	Lower limb	64 (54.7)	2	13	6	41	2
	Upper limb	36 (30.8)	0	4	1	28	3
	Other	7 (5.9)	0	Sacrum: 1	0	Groin: 1 Neck: 1 Buttock: 1 Back: 1 Trunk: 1 Head: 1	0
	Extra-cutaneous	10 (8.6)	Tongue: 1 Vulva: 1	Nasal cavity: 1 Anal: 1	0	Maxillary sinus: 2 Esophagus: 2^k^	Hard palate: 1 Epiglottis: 1
Clinical features^e^	Ulcerated	44 (44.9)	2	5	6	28	3
	Exophytic/ hyperkeratotic	36 (36.7)	0	3	1	30	2
	Verrucous, crusted or erosive	18 (18.4)	1	4	0	13	0
	NS	19	0	1	6	10	2
Size^f^ specified in 88 cases (75.2 %)	>2 cm <5 cm	19 (21.6)	0	1	1	15	2
	≥5 cm	33 (37.5)	1	5	3	22	2
	≤2 cm	36 (40.9)	0	0	0	34	2
	NS	29	2	12	3	11	1
cSCC duration^g^, specified in 42 cases (35.9 %) *n* = number of patient mean (months, mo) (median, range, mo)		42 9.7 (6, 1-36)	1 (4mo) NA NA	5 13.2 (7, 5-36)	4 14.8 (9, 5-36)	29 9.0 (6, 1-36)	3 5.3 (6, 2-6)
Histopathological Characteristics^h^ specified in 88 cases (75.2 %)	Well differentiated	65 (73.9)	1	10	7	44	3
	Moderately differentiated	16 (18.2)	1	2	0	11	2
	Poorly differentiated	7 (7.9)	1	0	0	5	1
	NS	29	0	7	0	21	1
Metastases, specified in 88 cases (75.2 %)	Loco-regional	14 (15.9)	1	0	0	10	3
	Visceral	20 (22.7)	0	2^l^	0	18	0
Death related to cSCC^i^, specified in 78 cases (66.7 %)		32 (41.0)	0	6	0	25	1
Relapse, specified in 72 cases (61.5 %)	Yes No	26 (36.1) 46 (63.9)	1 1	1 14	0 2	22 27	2 2
Mean time to recurrence (months), specified in 26 cases (22.2 %)		14.9	NA (1case) NS (2cases)	33 (6cases)	1 (1case)	9.5 (18cases)	1 (1case)

*DDEB* dominant dystrophic epidermolysis bullosa, *DEB* dominant epidermolysis bullosa, *EBS* EB simplex, *F* female, *JEB* junctional epidermolysis bullosa, *KS* Kindler syndrome, *M* male, *RDEB* recessive dystrophic epidermolysis bullosa, *RDEB-I* recessive dystrophic epidermolysis bullosa-Inversa, *NS* not specified, *NA* not applicable

^a^Six cases were reported in childhood or adolescence (range 6-17years), all of them affecting RDEB patients

^b^The mutated genes depending on EB type and subtype are defined in Table 4

^c^Family history was specified in only 38 cases (32.5 %)

^d^Except for one patient with JEB, cSCCs occurred in areas of chronic blistering, non-healing erosions/ulcerations or atrophic scarring. When a patient had several cSCCs, the most relevant location was taken into account

^e^Clinical features were specified in 98 cases (83.8 %)

^f^When for the same patient several cSCCs developed the one with the largest size was taken into account

^g^The time between the occurrence of cSCC and the confirmed diagnosis from a biopsy

^h^Unusual histological findings: verrucous cSCC (7 cases) and 2 angiosarcoma-like cSCC (2 cases). Depth beyond dermis, depth beyond subcutaneous fat, perineural invasion and lymphovascular invasion, were very rarely reported: depth mentioned in only 12 cases (8 RDEB, 2 JEB, 1 KS and 1 EBS) and perineural invasion in only 1 case (KS)

^i^Among these 78 cases, the death was related to SCC in 32 cases. The outcome of patients was not specified in 29.9 % (*n* = 35) of cases. Four patients were lost to follow-up

^j^ Sex ratio; ^k^One of the 2 cases concerned RDEB-I ^j^ (for the second case of RDEB-I cSSC was on the lower limb); ^l^ Lung metastases for one, NS for the other

The data available from molecular genetics analysis [5, 7–20] are presented both in Tables 3 and 4.

**Table 4:** Evolution of the disease in EB patients for whom diagnoses were confirmed by molecular analysis (*n* = 19)

EB type	EB subtype		Involved genes and mutations	Consequences	Age of cSCC diagnosis	Metastases	Death related to (c)SCC	Relapse	Time to recurrence (months)	Comments
EBS	EBS-DM		*KRT5* (*n* = 1) c.1431G > C ^38^	p.Glu477Asp ^38^	“mid-thirties"	no	NS	yes	“few months”	Verrucous leg carcinoma
	EBS-AR		*KRT14* (*n* = 1) c.1174G > T ^34^	p.Glu392Xaa ^34^	41	no	NS	NS	NS	SCC of the tongue
JEB	NH-JEB		*LAMB3* ^b^ (*n* = 5) c.628G > A + 1628G > A ^5^ c.29insC1628G > A ^5^ c.628G > A11903C > T ^5^ c.1903C > T + 1048A > C ^27^ c.29insC + 2500C > T ^39^ *COL17A1* ^c^ (*n* = 2) c.3236delC + 3236delC ^5^ 4003delTC ^12a^	p.Glu210Lys + Glu210Lys ^5^ p.Leu11ProfsX43 + Glu210Lys ^5^ p.Glu210Lys + Arg635Xaa ^5^ p.Arg635X + Thr350Pro ^27^ p.Leu11ProfsX43 + Gln834Xaa ^39^ p.Ser1079CysfsX26 + Ser1079CysfsX6^5^ NS ^12 a^	48 61 28 70 32 42 58	no no yes no yes yes no	no no yes no yes yes no	no yes yes NA yes yes no	NA 144 216 no 84 21 no	Death with lung metastases^5^ Follow-up period not specified^27^ Death with lymph nodes and lung metastases^39^ Death with lymph nodes and skin metastases^5^ Follow-up period not specified^12^
DEB	DDEB		*COL7A1* (*n* = 1) ^40^	p.Gly2079Arg ^40^	38	no	NA	NA	NA	Lost to follow-up
	RDEB	RDEB-HS	*COL7A1* (*n* = 3) c.5287C > G ^9^ c.6266_6269delCCCC ^*9*^ c.5797C > T ^41^ c. 5532 + 5G > A ^42^	p.Arg1753Xaa ^*9*^ Frameshift deletion resulting in a premature stop codon ^9^ p.Arg1933Xaa ^41^ Splice site mutation resulting in a 45-bp deletion ^42^	33 25 22	no yes NS	NS no NS	NS NA NS	NS NA NS	Lymph nodes metastases but death due to secondary amyloidosis ^41^
		RDEB-nHS	*COL7A1* (*n* = 3) c.238G > C ^13^ c.3631C > T ^13^ NS ^43^ 5818delC ^44a^	p.Ala80Pro ^13^ p.Gln1211Xaa ^13^ p.Glu2858Xaa and p.Gly2576Arg ^43^ p.Gly1815Arg ^44^	27 12 44	no no no	NS NS no	NS yes no	NS 9 NA	Sentinel lymph node performed and negative^13^ Follow-up period not specified^43^ Follow-up period of 3 years^44^
KS	NA		*FERMT1* ^*d*^ (*n* = 3) c.328C > T ^45^ c.1140-6 T > A ^46^	p.Arg110Xaa ^45^ Splice-site mutation ^46^	>60 (*n* = 2) 16	NS (*n* = 2) yes	NS (*n* = 2) yes	NS (*n* = 2) NA	NS (*n* = 2) NA	The patients were siblings^45^ Lymph nodes metastases (without histological proof)^46^

*AR* autosomal recessive, *DDEB* dominant dystrophic epidermolysis bullosa, *DEB* dystrophic epidermolysis bullosa, *DM* Dowling-Meara, *DNA* deoxyribonucleic acid, *EBS*, EB simplex, *JEB* junctional epidermolysis bullosa, *KS* Kindler syndrome, *HS* hallopeau-Siemens, *NH* non-Herlitz, n-HS non Hallopeau-Siemens, *RDEB* recessive dystrophic epidermolysis bullosa, *NA* not applicable, *NS* not specified, *KRT* keratin, *LAMB3* laminin subunit beta 3, *COL17A1* collagen type XVII alpha 1, *COL7A1*, collagen type VII alpha 1, *FERMT1* Fermitin family member 1, *KIND1* Kindlin-1. All of these genes are named according to the HUGO Gene Nomenclature Committee [47]

^a^We have chosen to write the mutation as it has been mentioned in the article (12,44), in order to do not misinterpret the data

^b^All of these patients had laminin-332 reduced in immunofluorescence, except for the patient from the Mohr et al. study [13] with laminin-332 negative

^c^These 2 patients were COL17A negative in immunofluorescence

^d^In the article of Arita et al. [20], the gene was named *KIND1*, but it is currently known as *FERMT1* according to the HUGO Gene Nomenclature Committee [47]

### Staging investigations

#### 18-Fluorodeoxyglucose Positron Emission Tomography/Computed Tomography (18-FDG PET/CT)

18-FDG PET/CT imaging was used in 5 cases (4 RDEB and 1 KS) [21–24]. An increase in FDG uptake was observed three times: twice, it was due to lymph node metastases (1 RDEB and 1 KS) confirmed by biopsy analysis, and once, to a non-specific inflammation. In two cases imaging was negative [7, 22].

### Sentinel lymph node

Four articles referred to the sentinel lymph node biopsy (SLNB) technique; all of them concerned RDEB patients. The procedure led to negative results in all cases. The outcome of these patients was not specified except for one, who was still alive 12 months after the procedure [8, 9, 25, 26].

### Therapy

#### Surgery

Most surgical approaches aimed at macroscopic clear margins, with subsequent histological confirmation. The size of the surgical margins was not mentioned in 96 cases. When they were specified (22 cases), they were between 1 and 3 cm.

In one case of hand cSCC in KS, radiotherapy was performed to shrink the tumor before local excision. It did not prevent local and regional recurrence and a forearm amputation with lymph node dissection had to be performed. Evolution of this patient was not specified, in particular it was not mentioned if death related to cSCC occurred.

Surgical amputation of at least a part of a limb was performed in 30 cases (23 RDEB, 2 DDEB, 2 JEB, 2 KS and 1 EBS). It concerned knee or below-knee amputation of the leg (*n* = 10), a toe (*n* = 1), a foot (*n* = 1), and above-knee amputation in other cases (*n* = 2). Hand, forearm and arm amputations were done in 9, 3 and 4 cases, respectively. Among these 30 amputees, 10 died due to metastatic disease progression. Except for one case of JEB-non Herlitz (JEB-nH), all were RDEB patients. Two were lost to follow-up, and 4 cases of RDEB were still alive (post-amputation monitoring from 24 months to 8 years). For the others cases (*n* = 14) the evolution was not specified. Concerning the JEB-nH patient, a skin biopsy from non-lesioned skin showed a plane of cleavage within the lamina lucida on transmission EM and reduced immunostaining with GB3 antibody, but mutational analysis was not performed [10].

Only 4 tumors were removed using microscopically controlled excision (Mohs technique). For the 2 RDEB-patients one had no relapse after 16 months of follow-up; while for the other patient the excision was incomplete and had to be followed by amputation. The two other cases concerned JEB patients. In the first case a relapse with distant metastasis was observed 1 year after surgery. The other patient had no new primary tumor or metastases after 31 months of follow-up.

### Systemic treatment

Systemic treatment was reported in 4 articles, representing 6 patients. Conventional chemotherapy was used for all; 3 patients received additional treatment with cetuximab.

Table 5 summarizes cases of cSCC treated by systemic treatment [21, 27–29].

**Table 5:** Systemic treatment of cSCC in EB patients

Reference (s)	Age (yr)/sex	EB subtype	Site of cSCC	Histological differentiation/size of tumor (cm)	Site(s) of metastases	Treatment	Outcome
Schwartz^16^	55 F	DDEB	Lower extremity (knee)	Well/>5	Not applicable	Intra arterial Doxorubicin + MTX	Surgical excision Survival unknown
Lentz^17^	22 F 26 F	RDEB	Upper extremity (forearm) Trunk	Well/unknown Unknown/>2 < 5	Axillary lymph nodes, pulmonary nodules Axillary lymph nodes	Cisplatin 5FU-cisplatin	PR Death from cSCC PR, surgical resection, alive at 12mo
Arnold^7^	24 F	RDEB	Upper (elbow) and lower (feet) extremities	Well/>5	Axillary lymph nodes	1.5FU-cisplatin 2. carboplatin-taxol 3. cetuximab	1. PR 2. PD 3. GR, alive at 3mo
Kim^18^	26 F 43 M	RDEB	Upper (hand) extremity Unknown	Moderately/>5 Well/>5	Axillary lymph nodes, pulmonary nodules Axillary lymph nodes, pulmonary nodules	1. cetuximab 2. cetuximab-gemcitabine 1. cetuximab 2. MTX orally 3. MTX IV	1. PD 2. Death from pneumonia 1. PD 2. Death from pneumonia

*cSCC* squamous cell carcinoma, *cm* centimeter, *DDEB* dominant dystrophic epidermolysis bullosa, *F* female, *GR* good response, *EB* inherited epidermolysis bullosa, *IV* intravenously, *M* male, *mo* month, *MTX* methotrexate, *PD* progressive disease, *PR* partial response, *RDEB* recessive dystrophic epidermolysis bullosa, *Yr* year, *5FU* 5 fluorouracil

### Radiotherapy and topical photodynamic therapy

Radiotherapy for cSCC was reported in 16 articles while topical photodynamic therapy was addressed in another, representing a total of 19 patients treated by these methods (Table 6). Specifically, twelve of these patients had RDEB. Half of the reported KS cases (*n* = 3) were treated with radiotherapy. Two cases were JEB. No case of cSCC occurred in EBS, and no case of DDEB was treated with radiotherapy. The total radiation doses ranged from 12 to 72 Gy (median 50.4 Gy). The total doses for tumor bed irradiation ranged from 45 Gy to 72 Gy (median 60.6 Gy). For nodal metastases total radiation doses ranged from 12 to 50.4 Gy (median 45 Gy). The tumor response did not appear grossly correlated with the total dosage. Tolerance of the treatment was not always reported. Five patients presented with grade 1 skin toxicity (3 RDEB, 1 KS and 1 JEB). Grade 2 cutaneous toxicity was observed in 1 RDEB patient and grade 3 in 2 RDEB patients [5, 8, 21, 29–41].

**Table 6:** Reported cases of (c)SCC, in EB patients treated by radiotherapy and topical photodynamic therapy

Reference (s)	Age yr/sex	EB subtype	Histological subtype of cSCC	Histological differentiation	Site(s) treated	Radiation delivery	Response	Survival (time before death)	Note (s)
Didolkar^19^	33 F	RDEB	Adenoacanthoma	Unknown	Sacrum	Cumulated dose = 60Gy	GR	Death (?)	Death due to hypercalcemia (and unknown metastasis)
Reed^20^	32 M	RDEB	Unknown	Unknown	Back	Unknown	GR	Death (3 years)	Death due to other cSCC
Keeff^21^	35 F	RDEB	Common	Well	Hand	3Gyx10 = 30Gy 3Gyx11 = 33Gy	PR then NR	Death (?)	
McGrath^22^	48 F	RDEB	Angiosarcomatoid	Unknown	Wrist Axilla	5Gyx9 = 45Gy 4Gyx3 = 12Gy	NR PR	Death (6months)	
Schreiber^23^	33 M	RDEB	Unknown	Unknown	Cervical lymphadenopathy	Unknown	Unknown	Unknown	
Bastin^24^	41 F 28 M	RDEB	Common Unknown	Well Unknown	Axilla Axilla	1.8Gyx32 = 57.6Gy 1.5x25Gy = 37.5Gy	PR then NR NR	Death (4months) Death (1month)	
Lotem^25^	34 F	KS	Common	Well	Hard palate	1.8x40Gy = 72Gy	CR	Alive at 2 years	
Weber^12^	26 F	RDEB	Common	Moderately	Leg and groin	Cumulated dose = 60Gy	PR	Death (7months)	
Mseddi^26^	18 F	RDEB	Common	Well	Groin	Cumulated dose = 45Gy	NR	Death (2months)	
Mallipedi^27^	45 M	JEB	Unknown	Unknown	Bladder	Unknown	Unknown	Unknown	Lost to follow-up
Souza^28^	51 F	RDEB	Bowen disease	Not applicable	Hand	Not applicable	CR	Alive at 2 years	PDT+ 5ALA
Emmanuel^29^	57 F	KS	Unknown	Moderately-poorly	Hand	Unknown	NR	Unknown	Neoadjuvant RTH
Arnold^7^	24 F	RDEB	Unknown	Well	Arm Axilla	Cumulated dose = 61.2 and 50Gy	PR	Alive at 3months	Remission probably due in part to systemic therapy^a^
Mituzani^30^	43 M	KS	Common	Well	Knee and epiglottis	Unknown	CR	Alive at 2.5 years	
Yuen^5^	55 M	JEB	Common	Unknown	Nasal cavity	Unknown	CR	Alive at 7months	
Onsun^31^	45 F	RDEB-I	Unknown	Well	Oesophagus	Unknown	NR	Death (8months)	
Kim^18^	26 F 43 M	RDEB	Common	Moderately Well	Axilla Axilla	Unknown	NR	Death (6months) Death (2months)	Despite systemic treatment^b, c^

*CR* complete response, *cSCC* squamous cell carcinoma, *F* female, *GR* good response, *EB* inherited epidermolysis bullosa, *JEB* junctional epidermolysis bullosa, *KS* Kindler syndrome, *M* male, *mo* month, *NR* non-response, *PDT* photodynamic therapy, *PR* partial response, *RDEB* recessive dystrophic epidermolysis bullosa, *RTH* radiotherapy, *Yr* year, *5ALA* 5 aminolevulinic-acid

^a^5FU-cisplatin then carboplatin-taxol then cetuximab

^b^cetuximab then cetuximab-gemcitabine

^c^cetuximab then methotrexate orally then methotrexate intravenously

## Discussion

Our systematic review of cSCC in EB gathered 117 cases. The most frequently published cases of cSCC arise in RDEB patients (81), followed by JEB (19), DDEB (7), and KS (7). cSCC in EBS (*n* = 3) appears as a rare event, at least rarely reported in the literature.

These data are consistent with the results of the analysis of the US National EB Registry showing that at least one cSCC occurred in 2.6 % (73/2745) of the study population [3]. The highest occurrence was noted in RDEB and JEB, with a frequency of cSCC, within their study population, of 50 % and 4.5 %, respectively. The lowest occurrence was noted in EBS and DDEB. KS was not mentioned in their study [3]. As to the gender, no difference was observed in the US National EB Registry nor in our study except for JEB with a sex ratio of 3.75:1 for men: women, suggesting, due to the lack of statistical data, that JEB occurs more frequently in men. Regarding ethnicity our study results contrast with those of *Fine et al.*, who found no difference. Our study showed a higher incidence of cSCCs in Caucasians (50.4 %) compared to that observed in Asians, Northern Africans and Hispanics [3]; however, 35.9 % of cases were not specified in our study and these differences could be due to reporting bias and should be interpreted objectively.

According to the literature cSCCs in EB patients occur at a much younger age (median age at diagnosis 36 years [IQR], 27-48 years and range, 6-71 years) than in non-EB patients (median age at diagnosis 80 years ([IQR], 73-86 years (range not available, [42]), and 71 years (range, 37-93 years (IQR not available, [43]). The youngest reported patient in our review was a 6-year-old girl with RDEB-nHS (non Hallopeau-Siemens). *Fine* et al. reported a cumulative risk in RDEB-HS (Hallopeau-Siemens) growing steeply from 7.5 % by age 20 to 67.8 %, 80.2 %, and 90.1 % by ages 35, 45, and 55, respectively. They described a cumulative risk of 0.8 % by age 14 for RDEB-nHS. In JEB-Herlitz (JEB-H), the risk was 18.2 % by age 25. The frequency of cSCC in their study cohort was surprisingly higher in JEB-H patients (4.4 %, i.e., 2 patients out of 45) than in JEB-nH patients (0.5 %) [3]. However, in another study [5], all cases of cSCCs occurred in JEB-nH, and the authors suggested that long term survival JEB-H patients might in fact be cases of JEB-nH. These results are in accordance with ours (Table 2).

The accurate appreciation of a difference in the risk of occurrence of cSCC between various types of EB should ideally be based on a molecular diagnosis of EB subtype in order to avoid misclassification. However, very few genetic data was available at the time of our EB and cSCC literature review: in summary, the diagnosis of EB was predominantly based on clinical features, IF and/or EM data, with less than 20 % of EB cases being confirmed by molecular analysis. Further, there was insufficient data for statistical analyses to attribute cSCC risk with each EB type with or without the molecular mutations, as was presented by Fine et al. [3]. In this article we provide some descriptive information about this topic, but once again it highlights the literature gaps and reiterates the need for more detailed studies and case reports/series.

Concerning data presented in Table 4, from JEB patients, all of them had laminin-332 reduced or negative in IF. Nevertheless, the lack of an epitope recognized by a laminin-332 chain monoclonal antibody does not necessarily mean there is a total absence of laminin-332. One of the main reasons is that total absence of laminin-332 almost always is lethal during infancy. Indeed, most often absence of staining with a monoclonal antibody is due to partial defects of laminin-332 caused by missense or deletion mutations which disrupt the epitopes recognized by the particular laminin antibody used. In our presented cases, the authors always used the monoclonal antibody GB3 which recognizes a conformational epitope that becomes disrupted even with subtle changes to the laminin-332 molecule.

The majority of cSCCs occurred on upper and lower extremities, particularly over bony prominences, and typically (99 % frequency) in areas of chronic non-healing ulceration(s). Indeed, only for one JEB-nH patient, cSCCs arose on non-affected skin. In our review, the occurrence on a sun-exposed area appears unusual and concerns only one case (RDEB-HS patient). The US National EB Registry reported 10 % and 100 % of cSCCs arising in a sun-exposed area in RDEB-HS and EBS patients respectively [3].

Although rarely reported, mucosal SCCs are possible and patients with dysphagia and/or dysphonia should be carefully examined. Importantly, one of the two cases of mucosal SCC occurring in an EBS-patients concerned a novel homozygous keratin 14 mutation in an autosomal recessive form of EBS (EBS-AR) [11]. Moreover, except for one case of esophageal SCC occurring in a non-smoking RDEB-I patient, no data are available concerning other potential risk factors (such as tobacco) for these extra-cutaneous locations. One explanation for mucosal SCCs in EB, could be the frequent and repetitive trauma to the mouth and throat from normal usage, resulting in cellular damage and then, over time, undifferentiated cancerous proliferation.

Our systematic review shows that it is difficult to evaluate prognostic factors in EB patients because of the frequent lack of use of *AJCC (American Joint Committee on Cancer*) *Tumour Staging for cSCC* [42, 43]. Prognostic factors such as poor differentiation, tumor diameter >2 cm, perineural invasion and invasion beyond subcutaneous fat, are rarely reported, rendering the staging difficult. Perineural invasion and the level of invasion are almost never mentioned (only 13 cases).

Our study did not allow exact assessment of the frequency of recurrence. Indeed, in several cases it was not clearly specified if the occurrence of local relapse or regional and/or distant metastases were synchronous with the diagnosis of cSCC.

Early detection of regional lymph node metastases can improve patient prognosis. Studies have demonstrated that SLNB can detect subclinical lymph node metastasis in patients with high-risk cSCC [44]. For EB patients such an invasive procedure may not always be performed due to their fragility. In addition, regional lymph nodes in these patients are frequently enlarged due to associated persistent inflammation or chronic infection. Thus, the risk of a false positive is considerable. The same analysis can be done with 18-FDG PET/CT. With only 4 and 5 articles on these topics, the specificity and sensitivity cannot be assessed. So, currently, data are too scarce to draw any conclusions about the interest of SLNB and 18-FDG PET/CT in the management of EB patients with cSCC. In clinical practice, their significance should be discussed case by case.

The treatment of choice is wide and deep surgical excision. Very recently Mellerio and coworkers recommended a 2 cm excision margin around the tumor, as assessed clinically. They specify that it is often difficult to define the tumor extent clinically and that the excision margin may be limited by anatomical considerations [6].

Clear excision margins do not always ensure a cure, and surgery is often an aggressive approach to treat large tumor in which wound healing is delayed. This is particularly true for RDEB due to the fragility of the surrounding skin. Amputation is often unavoidable: in our review, this was the case in a quarter of the EB patients with cSCC.

Neoadjuvant radiotherapy could be an option to decrease the tumor’s size and to minimize the surgery. Conversely, it is necessary to be very careful with this approach because of the potential toxicity to the surrounding skin. In our review, only one case (KS) had neoadjuvant radiotherapy but this did not prevent recurrence and disease progression.

In a few cases, Mohs micrographic surgery was performed. However, there is no evidence that this technique reduces morbidity and mortality as compared with conventional excision [3].

Radiotherapy for EB patients, in particular in generalized forms, can be complicated by poor wound healing and skin ulceration due to low skin tolerance to ionizing radiation. In our review, 7 of 20 (35 %) tumors displayed a measurable reduction of their size following radiation. For three patients a complete response was observed. These results show that radiotherapy may be beneficial in the palliation of EB patients with advanced disease. Another approach could be topical photodynamic therapy for Bowen’s disease as used by *Souza* et al. in RDEB [38]. Indeed, the prevention of invasive disease, which requires a potentially mutilating treatment, is very relevant. Nevertheless, this topic is controversial and many physicians would not encourage conservation treatment of Bowen's disease in RDEB.

Regarding systemic chemotherapy with cytotoxic agents in cSCCs, combinations of cisplatin with 5-fluorouracil (5-FU) or doxorubicin, have demonstrated some degree of efficacy [45]. Due to its toxicity, systemic chemotherapy is generally avoided for EB patients, and there are only four reports of its use in EB associated cSCC [21, 27–29]. *Lentz et al.*, showed good tolerance for two patients with RDEB; one of them was treated with cisplatin alone, the other one with a combination 5-FU-cisplatin, with no significant toxicity to the skin or oral mucosa. Both patients had partial clinical responses. For one of them with lung metastases, the evolution was marked by progressive metastatic disease and early death. For the other, with only lymph node metastases, a satellite lesion developed on the chest after the fourth course of chemotherapy requiring resection of the lesion and right axillary lymph node dissection. The patient was still alive 12 months after surgery [28]. In the study by *Fine et al.*, chemotherapy was used in 5.7 % of all RDEB patients. None experienced clinical benefits in terms of recurrence (data not shown) [3]. However, to establish any conclusions more data is required; systemic chemotherapy regimens could be palliative in this setting and should be interpreted objectively.

Single case reports and case series using the EGFR inhibitor cetuximab to treat patients with advanced or metastatic cSCC have been published [46]. For EB patients, *Kim et al.* used cetuximab as an adjuvant agent to surgery and radiotherapy in 2 RDEB patients. However, it did not prevent progression, leading to death [29]. *Arnold et al.* also tried cetuximab for one RDEB patient, after 2 lines of chemotherapy, and therapy was still ongoing after 3 months. However, this patient had only lymph node metastases without distant metastases [21]. The efficacy of using cetuximab in the treatment of advanced cSCC in EB patients has yet to be clarified. Its use as a neoadjuvant agent to surgery could be studied.

Our study complements the recent data published by *Mellerio* et al. [6]. In this systematic review the authors focused on the management of cSCC in EB-patients, and they established ‘best clinical practice’ guidelines. Interestingly and consistent with our data, most of their recommendations on “*surveillance and diagnosis, tumor evaluation and staging, surgical and non-surgical treatment, prostheses and end-of-life care*” have low levels of evidence, with a grade of recommendation D (D = evidence level 3; non-analytic studies, eg. case reports, case series or 4; expert opinion). Nevertheless, some good practice points were granted for the following items: the need for a multidisciplinary approach; interest of a lymphadenectomy in case of metastatic lymph node confirmed histologically; in some cases, a surgically aggressive approach such as amputation may be preferred to reduce recurrence risk; radiotherapy can be delivered but with caution, and in smaller fractions to minimize toxicity to surrounding skin; systemic therapy, with conventional chemotherapy or with target therapy, can be used palliatively taking into account the potential side effects; the crucial role of psychological support, both for the patients with EB and their family.

The main goal of our study was to detail, as exhaustively as possible, the cases of EB-patients with cSCC reported in the literature, and try to correlate and characterize this association. These results, as well as the results of those before us, must be viewed objectively because of the limitations of the analytic process. By its design, which incorporates data exclusively from single case reports or case series, this study cannot infer prevalence, incidence or risk of cSCC in the EB population. As EB is a rare disease, randomized trials or studies with a large number of patients are missing. When designing this study we were hoping to identify predictive factors of regional or distant metastases and/or death. We tried to see if sex, EB type and cSCC characteristics (location, size, clinical presentation and histological characteristics) could be predictive of disease progression, death and occurrence of metastases. Except for relapse and death due to cSCC which appear significant in the RDEB patients, we decided after an expert opinion and consultation with the statisticians, that these results were not relevant, whether in a univariate or multivariate analysis. In fact there was many missing data for each variable and the retrospective design implicated too many publications and biased reporting. For these reasons we did not incorporate any statistical analysis in this study.

## Conclusion

To conclude, our review confirmed that most of the cSCCs occurred in RDEB patients, but also emphasized that this common skin neoplasm has been reported in all EB types.

The first signs of cSCC develop at a younger age in EB patients than in non-EB patients. This study suggests and highlights the importance of regular monitoring of all patients, including those with EB. Furthermore, our work shows that when reporting cSCC cases in EB patients efforts should be made to: 1) better delineate the EB type to better understand risk; and 2) use AJCC Tumor Staging for cSCC to better define prognostic factors in these patients. As for other orphan diseases, randomized studies with a large number of subjects are difficult to perform. This highlights the important role of reference centres, to centralize all cases of EB and establish national registries to facilitate and promote clinical research in this area.

## Abbreviations

AJCC, American Joint Committee on Cancer; BCC, basal cell carcinoma; cSCC, cutaneous squamous cell carcinoma; DDEB, dominant dystrophic epidermolysis bullosa; DEB, dystrophic epidermolysis bullosa; EB, epidermolysis bullosa; EBS, EB simplex; EBS-DM, EBS-Dowling-Meara; EBS-K, EBS-Koebner; EBS-WC, EB simplex, Weber-Cockayne; EGFR, epidermal growth factor receptor; FERMT1, Fermitin family homologue 1; JEB, junctional epidermolysis bullosa; JEB-H, junctional EB, Herlitz; JEB-nH, JEB, non-Herlitz; KIND1, Kindlin-1; KRT14, keratin 14; KRT5, keratin 5; KS, Kindler syndrome; RDEB, recessive dystrophic epidermolysis bullosa; RDEB-HS, generalized RDEB, Hallopeau-Siemens; RDEB-I, RDEB-inversa; RDEB-nHS, generalized RDEB, non-Hallopeau-Siemens
